# An open source pipeline for quantitative immunohistochemistry image analysis of inflammatory skin disease using artificial intelligence

**DOI:** 10.1111/jdv.18726

**Published:** 2022-12-03

**Authors:** Yuchun Ding, Gaurav Dhawan, Claire Jones, Thomas Ness, Esme Nichols, Natalio Krasnogor, Nick J. Reynolds

**Affiliations:** ^1^ Interdisciplinary Computing and Complex Biosystems Research Group, School of Computing Science Newcastle University Newcastle upon Tyne UK; ^2^ Institute of Translational and Clinical Medicine Newcastle University Medical School Newcastle upon Tyne UK; ^3^ Department of Dermatology, Royal Victoria Infirmary Newcastle Hospitals NHS Foundation Trust Newcastle upon Tyne UK; ^4^ MRC/EPSRC, Molecular Pathology Node, Department of Pathology Newcastle Hospitals NHS Foundation Trust Newcastle upon Tyne UK

## Abstract

**Background:**

The application of artificial intelligence (AI) to whole slide images has the potential to improve research reliability and ultimately diagnostic efficiency and service capacity. Image annotation plays a key role in AI and digital pathology. However, the work‐streams required for tissue‐specific (skin) and immunostain‐specific annotation has not been extensively studied compared with the development of AI algorithms.

**Objectives:**

The objective of this study is to develop a common workflow for annotating whole slide images of biopsies from inflammatory skin disease immunostained with a variety of epidermal and dermal markers prior to the development of the AI‐assisted analysis pipeline.

**Methods:**

A total of 45 slides containing 3–5 sections each were scanned using Aperio AT2 slide scanner (Leica Biosystems). These slides were annotated by hand using a commonly used image analysis tool which resulted in more than 4000 images blocks. We used deep learning (DL) methodology to first sequentially segment (epidermis and upper dermis), with the exclusion of common artefacts and second to quantify the immunostained signal in those two compartments of skin biopsies and the ratio of positive cells.

**Results:**

We validated two DL models using 10‐fold validation runs and by comparing to ground truth manually annotated data. The models achieved an average (global) accuracy of 95.0% for the segmentation of epidermis and dermis and 86.1% for the segmentation of positive/negative cells.

**Conclusions:**

The application of two DL models in sequence facilitates accurate segmentation of epidermal and dermal structures, exclusion of common artefacts and enables the quantitative analysis of the immunostained signal. However, inaccurate annotation of the slides for training the DL model can decrease the accuracy of the output. Our open source code will facilitate further external validation across different immunostaining platforms and slide scanners.

## INTRODUCTION

The application of Artificial Intelligence (AI) is becoming increasingly prevalent in clinical pathology, clinical medicine and research. Within dermatological research, immunohistochemistry (IHC) and immunofluorescence (IF) techniques are commonly used to detect and quantify the presence of proteins in skin biopsy samples. Traditionally, these slides would be prepared and analysed manually, using commercially available or open‐source image viewing and analysis software. Sections can be analysed for various parameters including the presence of positive staining; the anatomical location of positive staining within the cell; the number of positively stained cells within the slide; and intensity of positive staining. As such, this process can be time‐consuming, labour intensive and can be prone to human error.

Deep‐learning (DL) are recognized computational methods that can identify patterns within large and complex datasets and generate classifiers of disease biology, clinical behaviour, or response to treatment. Therefore, a fully automated quantitative analysis using a DL model would address existing inefficiencies, improve service capacity and diagnostic efficiency, and support pathologists' decisions for research or service development.

Quantification of IHC/IF staining of certain proteins that distinguish normal from neoplastic tissue and/or provide prognostic information are increasingly being used as biomarkers in clinical practice. Multiple recent studies highlight the potential application of machine learning methods to quantify protein biomarker expression in IHC/IF slides.^1^, ^2^, ^3^, ^4^

There is, however, a relative paucity of studies examining the application of these methods to analyse IHC/IF in normal skin and inflammatory skin disease. Thus, for example, a PubMed search using the search terms “artificial intelligence AND immunohistochemistry AND skin” in August 2021 revealed only 15 results.

In this study, we aimed to explore if the routine (manual) image analysis and quantification process can be made more efficient and consistent by the application of an AI‐assisted analysis process for the quantification of IHC/IF stained skin sections. There are multiple proteins of interest in the context of on‐going studies in atopic eczema and psoriasis that we are currently studying using IHC/IF. For this article, we have focused on proteins that show distinct distributions across epidermal and dermal compartments and localize predominantly to the cytoplasm, cell membrane or nucleus. The primary endpoint we have selected for this study is the quantification of the ratio of positively stained cells within the epidermis and dermis in the disease state and post‐treatment.

## METHODOLOGY

### Subject recruitment and tissue samples

Skin punch biopsies (4–6 mm) were taken from the lower back/buttock of eight psoriasis and six atopic eczema patients at the Royal Victoria Infirmary, Newcastle Upon Tyne (RVI) following informed consent, regional research committee approval and in accordance to the declaration of Helsinki principles. Biopsies were bisected and one half fixed overnight in formalin solution (4% in PBS) and then embedded into paraffin blocks .

### Automated immunostaining details

All immunohistochemistry (IHC) was performed on the automated Roche Ventana Discovery Ultra platform using Ventana staining reagents. Brightfield DAB staining was performed with DISCOVERY® ChromoMap DAB Kit (RUO). Catalogue number 760‐159. Secondary antibodies used were anti‐mouse (product code 760‐4310) or anti‐rabbit (760‐4311) (dependent on primary antibody species) OmniMap HRP multimers. The panel of immunostaining markers (Table 1) was chosen to cover a variety of labelling patterns including membrane, cytoplasmic and nuclear staining. All antibodies detailed in Table 1, were optimized on the automated Ventana Discovery Ultra using recommended parameters from the manufacturers.

**TABLE 1:** Antibodies used for immunostaining

Antibody	Clone	Concentration	Manufacturer	Product reference
Cyclin B1	Rabbit monoclonal Y106	1:50	Abcam	Ab32053
Cyclin D1	Rabbit monoclonal SP4‐R	Pre‐dilute	Ventana	790‐4508
Phospho‐Histone H2AX	Mouse monoclonal 3F2	1:6000	Invitrogen	MA1‐2022
KI67	Mouse monoclonal MIB‐1	1:100	Dako	M7240
Kallikrein 7	Rabbit polyclonal	1:50	Abcam	Ab96710
Claudin	Rabbit monoclonal SP128	1:800	Abcam	Ab115783
Periplakin	Rabbit monoclonal EPR8296	1:1000	Abcam	Ab131269
NFAT5	Rabbit polyclonal	1:50	Thermo Fisher Scientific	PA1 023
Loricrin	Rabbit monoclonal EPR7148(2)	1:500	Abcam	Ab176322
Filaggrin	Mouse monoclonal 15C10	1:100	Leica	NCL Filaggrin

### Scanning of immune‐stained slides

Multiple slides were scanned at a time using the AutoLoader feature of the Aperio AT2 slide scanner (Leica Biosystems). Slides were placed into racks then inserted into the scanner. Snapshots were taken of all slides and then focus points were adjusted and/or added to each section on every slide. Slides were then scanned at 40× magnification.

### Annotating tissue/cellular structure in QuPath

One of the most commonly used tool boxes for viewing and analysing whole slide images is QuPath, an open‐source software that provides an intuitive interface that is straightforward to use.^5^ The slides were first imported to QuPath and then manually labelled. Six eczema and eight psoriasis patients with 97 and 96 sections, respectively, were annotated. Sections of psoriasis and eczema were immunostained with claudin, cyclin B1, cyclin D1, filaggrin, γ‐H2AX, kallikrein 7, ki67, loricrin, NFAT5 and periplakin. Immunostaining with claudin, cyclin B1, cyclin D1, filaggrin, γ‐H2AX, kallikrein 7, ki67, loricrin, NFAT5 and periplakin was confined to eczema slides and immunostaining with cyclin B1, cyclin D1 and γ‐H2AX was confined to psoriasis slides. At least two slides per immunostain for eczema were annotated. Annotation was performed on nine cyclin B1 slides, eight cyclin D1 slides and six γ‐H2AX slides. The annotations are then exported from QuPath in a form of image masks (Figure S1) and divided into smaller image blocks (for example 700 pixels by 700 pixels). This resulted in more than 4000 image blocks. This process was introduced because of the large size of the whole slide images which are too large to be used for deep learning directly. In addition, because the intensity of IHC stains varies across different slides, image normalization is applied to each of the image blocks to reduce variation in colour as described.^6^

### Implementation of AI

Deep learning is a type of artificial intelligence which imitates the neural network of the human brain to predict an outcome through continually learning input data (in this study, digital pathology images). Practically, this offers the potential for automatic identification of those objects and structures that must discriminate between different biological or clinical phenotypes. For this study, we used Deeplab v3+, a well‐known open source deep learning toolbox. Specifically, Deeplab v3+ is a type of CNN, 18 layers deep that is designed to improve the segmentation of the boundaries and to handle the problem of segmenting objects at multiple scales, by employing an encoder‐decoder layer which takes into account the essential information from the input image. It has been previously trained on a large dataset of regular objects to classify images into 1000 object categories, such as trees and animals, to learn rich feature representations for a wide range of images, which is suitable for tasks with limited processing resources. In this study, Deeplab v3+ was refined using annotated images in MATLAB to differentiate different histology features.^7^, ^8^, ^9^ In brief, the annotated data was fed to the pre‐trained AI model, and the AI made a prediction about what that data was (e.g. epidermis). The error occurring in the prediction was used to update the strength of the connections within the neural networks. This process continued until the AI was making predictions with sufficient accuracy.

**FIGURE 1:**
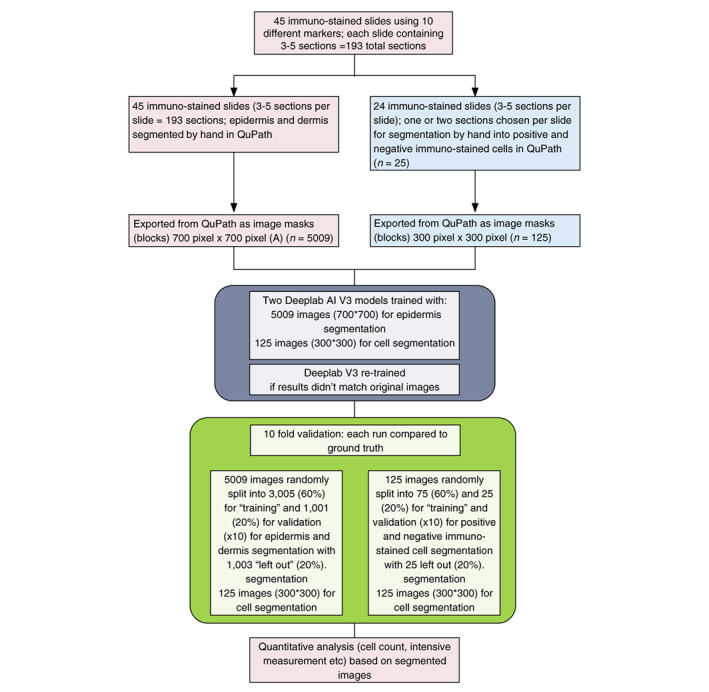
Study flow chart for training of Deeplab V3 AI. Numbers of immunostained slides, sections and images derived are provided for segmentation of epidermis and dermis on the left and cell segmentation on the right. The two Deeplab AI V3 models were run sequentially and then validated using random sampling to provide quantitative outputs and comparisons to ground truth images.

Two deep learning models (algorithms) were trained and applied in sequence: the first was used to label epidermis and the second was used to label negatively and positively immunostained cell. For segmentation of the epidermis from dermis, we used 5009 images (700 by 700 pixels) and for segmenting positively labelled from negatively labelled immunostained cells 125 images (300 by 300 pixels) were used in total (Figure 1). The deep learning models were trained using two Nvidia GTX 2080 TI GPUs (Graphics Processor Unit). That is, the models did not need expensive and specialized high‐performance clusters to be trained.

Specifically, the first algorithm coursed through the entire slide, region by region, (700 by 700 pixels at 20× magnification for epidermis segmentation, 300 by 300 pixels for cell segmentation) from top left to bottom right and applied semantic segmentation specifically to areas of the slide where specimens were located. The output of this implementation resulted in two grayscale images (Figure S2).

In the output image for segmentation of epidermis, 1 represents epidermis; 2 represents dermal papilla; 3 represents hair follicle, sweat gland and other artefacts; 4 represents the background. The images are binarized to produce a mask where 1 is epidermis and 0 is any other structure. Artefacts that could compromise the accuracy of the analysis were still present in some segmentation images. Due to the complexity of the structure, these artefacts were removed manually. In part, this relates to the integrity of the tissue sample and therefore adds an element of unpredictability to the workflow that would require manual review for subsequent analyses.

In the output image for segmentation of cells, 1 represents the positive cell membrane, 2 represents negative cell membrane, 3 represents positive cell nucleus, 4 represents negative cell nucleus and 5 represents other non‐cell structure. Because of the time and logistical constraints of labelling each cell in a section, the training dataset (in terms of numbers of sections) for cell segmentation was relatively small (see next paragraph). Therefore, we further enhanced the performance by implementing adaptive image thresholding, a method that can define the optimal thresholding value automatically, on the grayscale image of the original slide where 1 represents the positive stain and 0 represents the other structure. The two images were then combined and binarized where 1 is positive cells, 2 is negative cells and 0 is any other structures.

Segmented objects, such as noise (image object with an overall size of ≤100 pixels) were then removed. A previously described watershed‐based method was then applied to the binary image to split clustered cells.^10^ Additionally, at the start of the work‐flow, the AI algorithms were re‐trained when discrepancies to the ground truth were observed and if needed amendments to labelling and/or inclusion of further samples.

Finally, the images were post‐processed to produce quantification of cell ratio (positive/negative) within epidermal regions.

### Validation of AI

A validation process was performed to quantify the performance of AI methodologies. For epidermis segmentation, 3005 image blocks (700 by 700 pixels) comprising all the immunostains (Table 1) were randomly selected for training the deep learning model; 1001 were used for testing and 1003 were left out (Figure 1). This process was repeated 10 times to flag problems such as overfitting or selection bias. Each run was compared with each other and an average outcome determined. For cell segmentation, 75 image blocks (300 by 300 pixels) were randomly selected for training the deep learning model, 25 were used for testing and 25 were left out (Figure 1).

Transfer learning was applied to fine‐tune the trained models on a smaller dataset that involved only images with γ‐H2AX cases (10 slides, 44 sections) (Figure S3). The validation process was performed on 560 image blocks for epidermis and dermis segmentation (392 [70%] for training, 140 [25%] for testing and 28 [5% left out] and on 60 image blocks for single marker cell segmentation [42 for training], 15 for testing and three left out on each run).

### Statistical analysis

The numbers of positive and negative cells as well as the average signal and percentage of positive cells are shown as medians and interquartile ranges. Statistical analysis was performed using GraphPad Prism version 9.0.0, GraphPad Software, LLC using Mann Whitney U test.

## RESULTS

**FIGURE 2:**
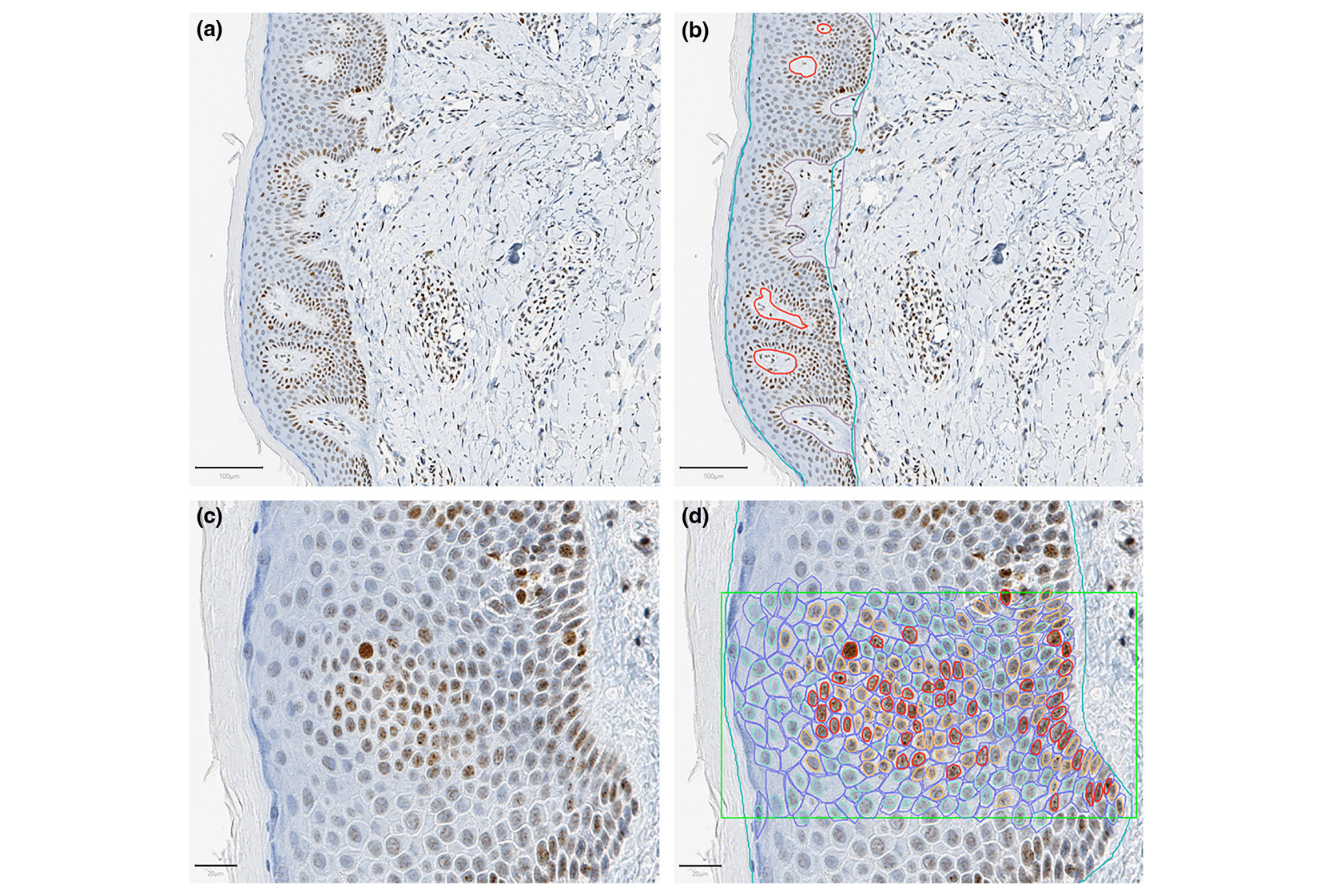
Using QuPath to annotate the epidermis, cellular structures and positively and negatively stained cells within lesional eczema skin. (a) Representative image of γ‐H2AX immunohistochemical staining. Magnification = 12×, scale bar = 100 μm. (b) Representative image of γ‐H2AX immunohistochemical staining with manual annotations for AI training. Epidermis labelled in blue, dermal papilla in purple and cross section dermal papilla in red. Magnification = 12×, scale bar = 100 μm. (c) Higher magnification representative image of γ‐H2AX immunohistochemical staining. Magnification = 40×, scale bar = 20 μm. (d) Higher magnification representative image of γ‐H2AX immunohistochemical staining with manual annotations for AI training. Box within which cells were annotated labelled in green. Strong positive cells labelled in light blue with strong positive nuclei labelled in red. Weak positive nuclei labelled in orange and negative nuclei labelled in turquoise. Magnification = 40×, scale bar = 20 μm.

Before analysis by AI, images were manually annotated in QuPath. In this study, we applied two sets of annotation which were colour‐labelled. The first category delineated and identified tissue category (e.g. epidermis) and artefact (Figure 2). The second category delineated and identified positively and negatively immunostained cells within the tissue (Figure 2).

### Implementation of AI

Once a DL model is trained, it can be used to classify every pixel in a whole slide image and produce an image that is segmented by class. For this project, two deep learning models were trained and applied in sequence. The first was used to label tissue areas, and the second was used to label negative and positive immunostained cells. The DL model for the detection of tissue areas, specifically epidermis was trained on 45 immunostained tissue slides comprising 3–5 tissue samples (resulting in more than 4000 image blocks). The algorithm for detecting positive areas of immunostaining, positive and negative labelled immunostained cells and nuclear regions was trained across 24 immunostained tissue slides comprising 3–5 tissue samples and 10 immunostains (Figure 1). During the development process, we compared the output of the Deep Learning algorithms with the ground truth images. If the results from the DL model did not closely match the original images, then further annotation and training of the model was done. This process underscored the importance of accurate annotation for training. The final model was independent of the immunostain.

**FIGURE 3:**
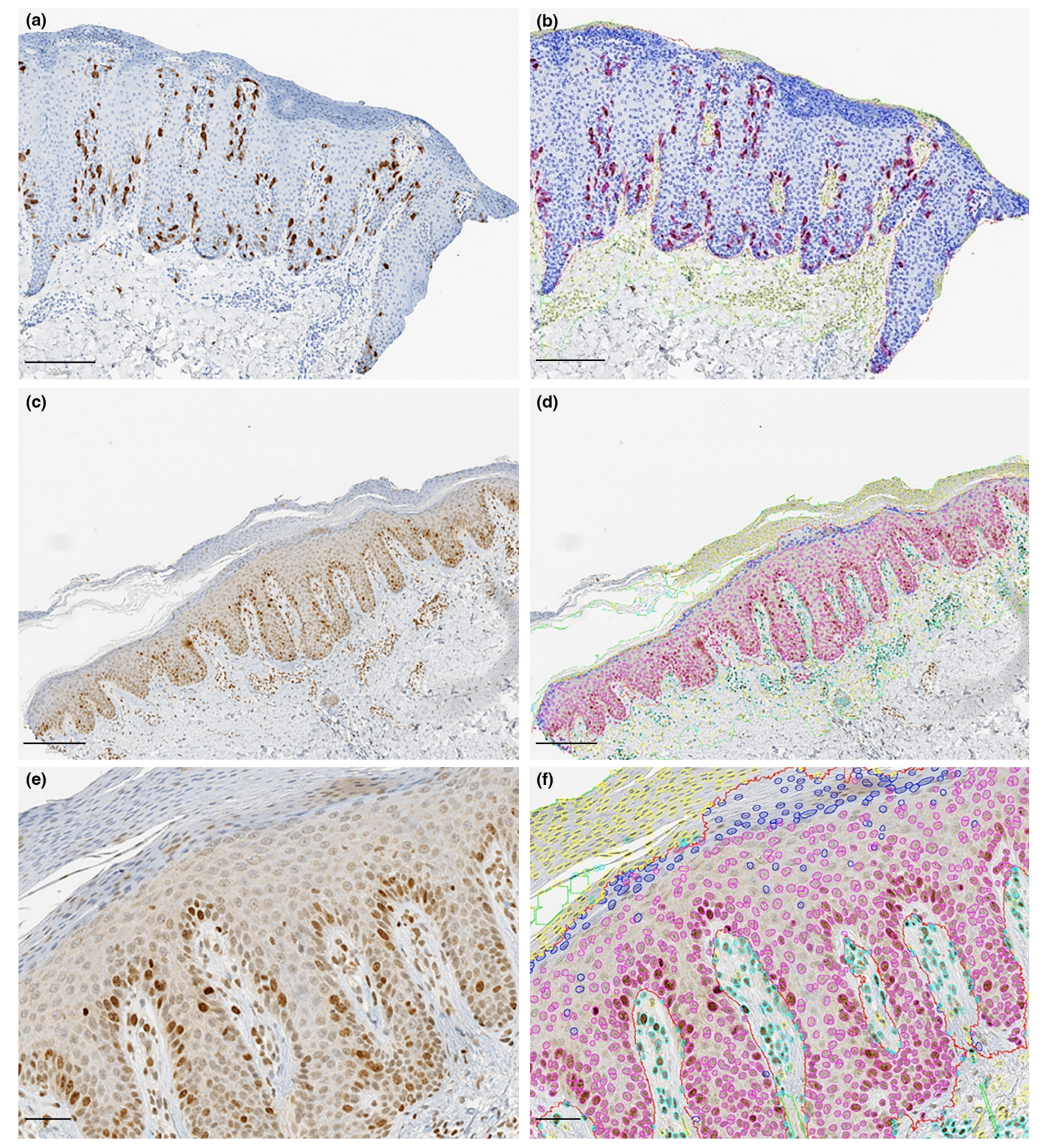
Representative image outputs from the deep learning models. (a) Representative image of cyclin B1 immunohistochemical staining in psoriatic lesional skin. Magnification = 6×, scale bar = 200 μm. (b) Image outputs from the trained deep learning (DL) model for slide (a). Magnification = 6×, scale bar = 200 μm. (c) Representative image of γ‐H2AX immunohistochemical staining in psoriatic lesional skin. Magnification = 6×, scale bar = 200 μm. (d) Image outputs from the trained DL model for slide (c). Magnification = 6×, scale bar = 200 μm. (e) Slide (c) (representative image of γ‐H2AX immunohistochemical staining in psoriatic lesional skin) at higher magnification. Magnification = 15×, scale bar = 50 μm. (f) Image output from the trained DL model on slide (e) (representative image of γ‐H2AX immunohistochemical staining in psoriatic lesional skin) at high magnification. Magnification = 15×, scale bar = 50 μm. Epidermis (red), positive cells inside epidermis (purple), negative cells inside epidermis (blue), positive cells outside epidermis (cyan) and negative cells outside epidermis (yellow).

Representative results of the outputs from the trained DL models are shown in Figure 3.

### Validation

The validation process involved sampling, training and testing the dataset randomly for 10 times was performed to evaluate the performance of The DL model for the segmentation of tissue areas. Several measurements, which are commonly used to evaluate the performance of semantic segmentation algorithms, were produced.^11^ As indicated in Figure 1, 5009 images were randomly split into 3005 (60%) for “training” and 1001 (20%) for validation for epidermis and dermis segmentation with 1003 “left out” (20%). Similarly, 125 images were randomly split into 75 (60%) and 25 (20%) for “training” and validation for positive and negative immuno‐stained cell segmentation with 25 left out (20%) (Figure 1).

The GlobalAccuracy represents the percentage of correctly classified pixels [(True Positive + True Negative)/Total Pixel]. MeanAccuracy [True Positive/(True Positive + False Negative)] indicates the percentage of correctly identified pixels for each class. Intersection over union (IoU) computes the intersection of the output image and the ground truth divided by the union of both images. This is also known as the Jaccard similarity coefficient.^11^ MeanIoU represents (True Positive)/(True Positive + False Positive + False Negative). WeightedIoU is the average IoU of each class, weighted by the number of pixels in that class. Finally, Mean Boundary F1 (BF, contour matching) Score,^11^ indicates how well the predicted boundary of each class aligns with the true boundary.

The results of each validation run and the average of the 10 runs are presented in Table 2. These data showed an average GlobalAccuracy of 94.95%, representing the percentage of correctly identified pixels for each class (i.e. epidermis). When weighted for the number of pixels in that class (epidermis) (WeightedIoU), this showed an accuracy of 90.50%.

**TABLE 2:** Performance analysis of deep learning (DL) model for segmentation of epidermis

Epidermis	GlobalAccuracy	MeanAccuracy	MeanIoU	WeightedIoU	MeanBFScore
Fold 1	94.99	92.99	87.91	90.54	71.23
Fold 2	95.28	94.3	88.58	91.14	72.38
Fold 3	94.89	93.28	87.68	90.4	70.69
Fold 4	94.55	92.74	86.86	89.8	70.07
Fold 5	95.09	93.68	87.98	90.79	72.03
Fold 6	94.48	92.83	87.11	89.64	69.14
Fold 7	95.13	93.45	88.25	90.8	71.51
Fold 8	94.98	93.25	87.74	90.57	69.97
Fold 9	94.85	93.33	87.56	90.34	71.06
Fold 10	95.18	93.15	88.08	90.9	71.91
Average	94.9513	93.1593	87.708	90.5033	70.8227

A similar validation process was also applied to quantify the performance of The DL model for segmentation of positive/negative cells. The results of each validation run and the average of the 10 runs are presented in Table 3. These data showed an average GlobalAccuracy of 86.09%, representing the percentage of correctly identified pixels for each class (i.e. positive and negative cells). When weighted for the number of pixels in that class (positive cells) (WeightedIoU), this showed an accuracy of 77.74%.

**TABLE 3:** Performance analysis of deep learning (DL) model for segmentation of positive/negative cells

Cell	GlobalAccuracy	MeanAccuracy	MeanIoU	WeightedIoU	MeanBFScore
Fold 1	83.62	70.17	57.75	74.19	67.95
Fold 2	88.39	73.58	61.94	81.28	75.03
Fold 3	85.48	69.63	58.28	76.79	74.04
Fold 4	85.27	67.79	57.38	76.12	72.75
Fold 5	84.51	69.74	59.02	74.56	69.98
Fold 6	87.42	72.44	60.42	79.99	74.45
Fold 7	85.32	68.97	57.81	76.53	72.25
Fold 8	83.64	70.22	57.79	74.21	68.02
Fold 9	88.37	73.59	61.91	81.26	75.07
Fold 10	85.48	69.63	58.29	76.8	74.04
Average	86.09867	70.722667	59.222	77.7433333	73.29

**FIGURE 4:**
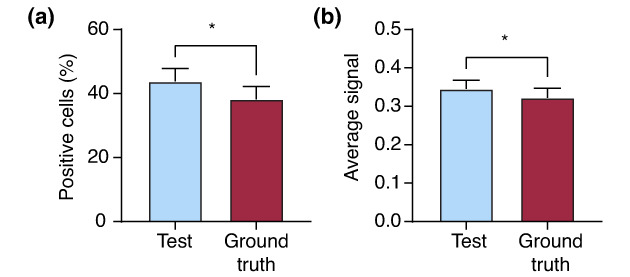
Quantitative analysis of deep learning (DL) model used to label negative and positive immunostained cells compared with ground truth (manually annotated images). (a) Percentage of positive cell and (b) Average signal. *p<0.05, unpaired t‐test.

In addition, we performed a quantitative analysis for the negative and positive immunostained cells, based on the segmented images using the DL model versus manually labelled images, which is shown in Figure 4.

The data has shown that the number of positive and negative cells is less accurate compared with the percentage of positive cells and average signal. This is because the dataset contains images that involve mixed staining protocols, e.g. images with a nuclear and cytoplasmic immunostained signal, which resulted in lower segmentation accuracy. Therefore, we used transfer learning to fine‐turn the trained models on a smaller dataset that involved only images with γ‐H2AX cases (nuclear immunostained signal). Specifically, 560 image blocks for epidermis segmentation and 60 image blocks for cell segmentation. Additionally, the validation process was performed to quantify the performance of the refined γ‐H2AX DL model. The results of each validation run and the average of the 10 runs are presented in Tables 4 and 5. These data showed an average GlobalAccuracy of 94.08% for the segmentation of epidermis and a significantly improved accuracy of 91.01% for the segmentation of cells.

**TABLE 4:** Performance analysis of deep learning (DL) model for segmentation of epidermis with γ‐H2AX stains

Epidermis	GlobalAccuracy	MeanAccuracy	MeanIoU	WeightedIoU	MeanBFScore
Fold 1	94.47	93.89	88.8	89.55	64.6
Fold 2	94.46	93.85	88.54	89.55	64.38
Fold 3	94.71	93.93	88.82	90	65.21
Fold 4	94.48	93.85	88.73	89.56	65.78
Fold 5	94.26	93.71	88.09	89.22	63.43
Fold 6	94.28	93.53	88.34	89.2	63.44
Fold 7	94.04	93.27	87.66	88.79	63.98
Fold 8	94.34	93.58	88.29	89.32	64.42
Fold 9	94	93.35	87.59	88.74	62.01
Fold 10	94.34	93.54	88.31	89.31	64.46
Average	94.0867	93.306	87.7613	88.872	63.2647

**TABLE 5:** Performance analysis of deep learning (DL) model for segmentation of positive/negative γ‐H2AX cells

Cell	GlobalAccuracy	MeanAccuracy	MeanIoU	WeightedIoU	MeanBFScore
Fold 1	88.32	83.01	71.33	80.15	70.23
Fold 2	90.27	83.47	72.82	83.23	69.24
Fold 3	90.75	83.57	72.83	84.09	68.17
Fold 4	91.35	82.91	71.97	85.24	67.89
Fold 5	90.15	83.12	72.21	83.12	70.48
Fold 6	90.38	82.36	71.23	83.63	65.26
Fold 7	91.04	82.37	71.66	84.66	67.23
Fold 8	90.5	82.28	71.77	83.67	65.91
Fold 9	89.73	82.48	71.72	82.38	67.9
Fold 10	91.12	81.11	69.01	85.26	62.36
Average	91.01	81.572667	70.462	84.7666667	64.1767

## DISCUSSION

To date, the study of AI‐assisted image analysis in skin pathology and has largely focused on cancer diagnosis. In this article, we describe the development, workflow and validation of an AI‐assisted analysis pipeline to segment and quantitate immunostained skin samples derived from inflammatory skin disease. We describe the process for annotating whole slide images of skin biopsies, immunostained with a variety of markers using automated technology. The application of two DL models in sequence facilitates accurate segmentation of epidermal and dermal structures, exclusion of common artefacts and enables the quantitative analysis of cell membrane, nuclear and cytoplasmic immunostained signals. Quantification of immuno‐stained sections of skin is increasing required for the development and validation of biomarker signals as potential co‐diagnostics for endotype definition, in the field of precision medicine, for example. By openly reporting methodology and publishing the code used to create AI algorithms (which does not require specialist high‐performance computing clusters to train), we support (a) the immediate application of our AI‐assisted model in the study of inflammatory skin disease; (b) the onward development of AI and its application to the analysis of skin histology, pathology and quantitative analysis of immunostained sections in clinical practice and (c) the potential to apply and extend the principle AI‐assisted features to other classes of skin disease.

Inaccurate segmentation of the tissue/cellular structure may potentially invalidate the subsequent quantitative signal analysis, including assessment of nuclear and cytoplasmic ratio and distribution across epidermis and dermis. This is typically caused by a lack of accurate annotation for training the deep learning model, but can be caused by the following reasons:

1. Lack of accurate annotation due to human‐error (Figure S4). This can be improved by feedback and improvement of annotating skills.
2. Unwanted artefact with similar appearances to the targeted tissue structure (Figure S5). Such artefacts can be removed manually or by re‐training the DL model with an additional tissue category as “other tissue categories” or “artefact”.
3. Inconsistent image features that confuse the AI (Figure S6). This is usually due to the involvement of multiple staining protocols or whole slide scanners from different manufacturers.^12^ Hence, we utilized the rigorous and automated immunostaining protocols developed in the Newcastle MRC/EPSRC Molecular Pathology Node and ensured all slides were scanned at ×40 magnification on one slide scanning machine with unified scanning settings. Alternatively, a further increase in manual annotations can be used to train DL models to adapt and can overcome the variation in immunostaining protocols and/or the use of different slide scanners. However, for the development of consistent AI protocols and short‐term studies that involve smaller datasets, it is highly recommended to maintain a consistent lab protocol for immunostaining and use of the same whole slide scanner for slide digitization.

There is a relative paucity of studies looking at the application of DL methodologies to develop AI‐assisted tools to analyse images of IHC/IF skin slides with a focus on segmentation of morphological compartments and quantification of positive staining.

Sakar et al.^13^ used a trained feedforward neural network to analyse morphological patterns of IF staining with a correct pattern of staining identified first time in 83% of slide. With regards to the accuracy of segmentation, our AI algorithm showed a global accuracy of 95%. This compares favourably to other AI algorithms such TuPaQ used by Abdelsamea et al.^14^ which showed a sensitivity of 84% in segmenting epithelium and non‐epithelium in colorectal sections. Our other main outcome was accurate quantification of positive immunostaining in each compartment in our samples and the AI algorithm achieved a GlobalAccuracy of 86.1%. This is broadly comparable with other AI algorithms in the literature such as U‐Net which was used to identify CD3 and CD8 positive lymphocytes in breast, colon and prostate cancer tissue slides with an F1 score (the harmonic mean of sensitivity and positive predictive value) of 0.78.^15^

However, when our model was fine tuned further using the smaller data‐set including only one immunostain (γ‐H2AX) this further improved GlobalAccuracy for quantification of positive immunostaining to 91.01% compared with manual annotation. Although this was a relatively small dataset including only 60 image blocks this data suggests that our model could be further refined to on a stain specific basis to provide accurate quantification of positive staining in skin biopsy slides.

The results of this proof of concept study show the AI‐assisted model to be effective at segmenting epidermis and dermis and quantifying positive IHC/IF staining in inflammatory skin disease compared with manual methods. We envisage that at this stage, the model's main application will be in the research setting to provide accurate and time‐effective quantification of immunostaining. However, as biomarkers of inflammatory skin disease move towards clinical application and as AI‐models develop, we envisage such models will enter clinical practice. Traditionally, segmentation of tissue compartments and quantification of IHC/IF staining would be done manually by microscopy. This is time consuming and prone to human error. The use of whole slide scanning and AI to automate these processes serves to improve efficiency and accuracy. Additionally, a dermatopathologist is not required with our approach as research associates can be trained to segment tissue sections and identify selected IHC/IF positive staining. Whole slide imaging systems have been shown to be non‐inferior when compared with microscopy.^16^ Similarly studies have demonstrated high concordance between digital pathology systems and manual pathology for detection and quantification of positive immunostaining.^17^, ^18^ However, there is limited literature to support the use of digital pathology and AI models in dermatopathology and dermatological research. We recognize the limitations of our study including small sample sizes for the training and validation of the AI; however, we believe the results show promise and warrant further refining of the AI model and so by making the computer code open source, we encourage the sharing of methodologies, their wider application and subsequent improvement in algorithm development.

## FUNDING INFORMATION

This work was supported by Medical Research Council (MRC) grants—Newcastle MRC Molecular Pathology Node (MR/N005872/1), Confidence in Concept to Newcastle University (MC_PC_17168), The Rosetrees Trust and the Newcastle National Institute for Health Research (NIHR) Biomedical Research Centre. Nick J. Reynolds is a NIHR Senior Investigator and is also supported by the NIHR Newcastle In Vitro Diagnostics Co‐operative.

## CONFLICT OF INTEREST

YD is a director of XWOW Ltd., a social enterprise company. NJR reports grants from PSORT industrial partners as listed (http://www.psort.org.uk/); other research grants from GSK‐Stiefel and Novartis; and other income to Newcastle University from Almirall, Amgen, Janssen, Novartis, Sanofi Genzyme Regeneron and UCB Pharma Ltd. for lectures/attendance at advisory boards. The other co‐authors have no disclosures.

## Supporting information


Figure S1

Figure S2

Figure S3

Figure S4

Figure S5

Figure S6

## Data Availability

The open source computer code utilized in this manuscript is available for download at https://github.com/khcy82dyc2/SkinAI.
