# Supplementary figures and images for: An open source pipeline for quantitative immunohistochemistry image analysis of inflammatory skin disease using artificial intelligence
Source: J Eur Acad Dermatol Venereol. 2022 Dec 3;37(3):605–14. doi: 10.1111/jdv.18726 (PMC10947200; doi:10.1111/jdv.18726)

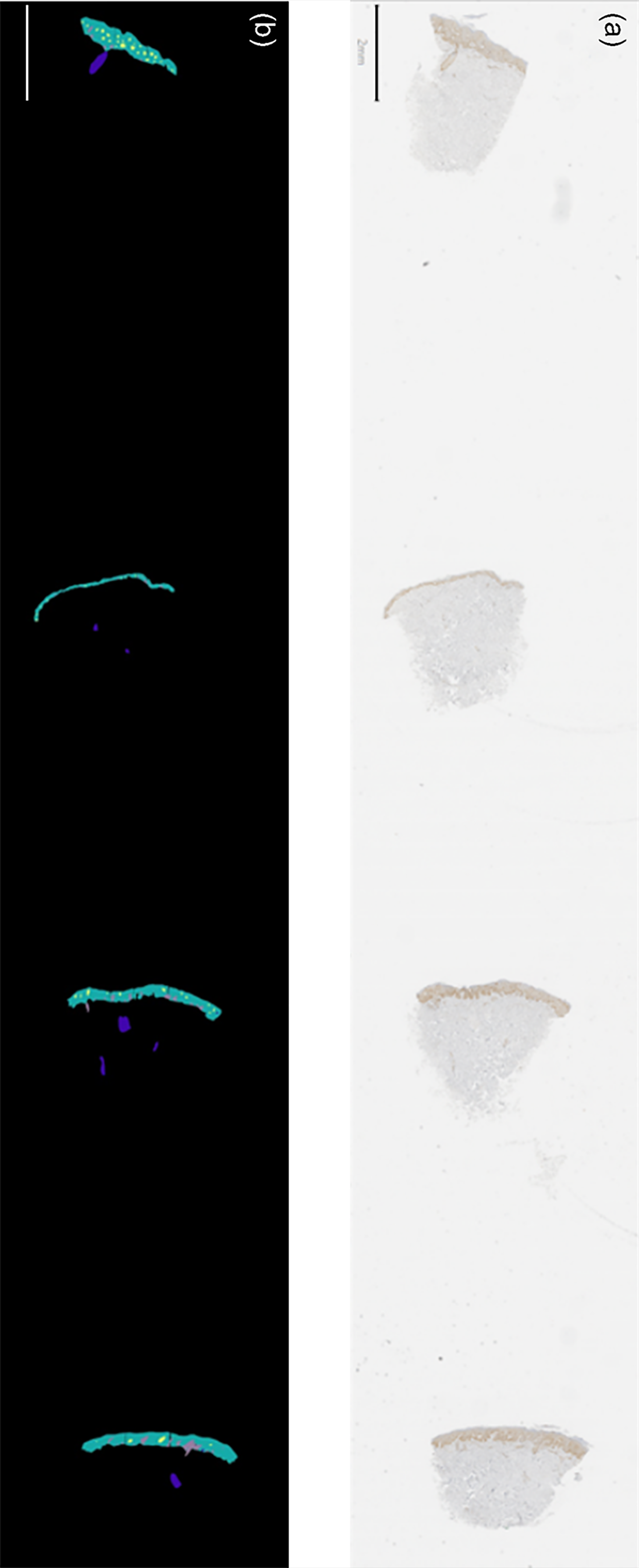

Supplement: Supplementary file 1 — Figure S1 [file JDV-37-605-s002.tiff]

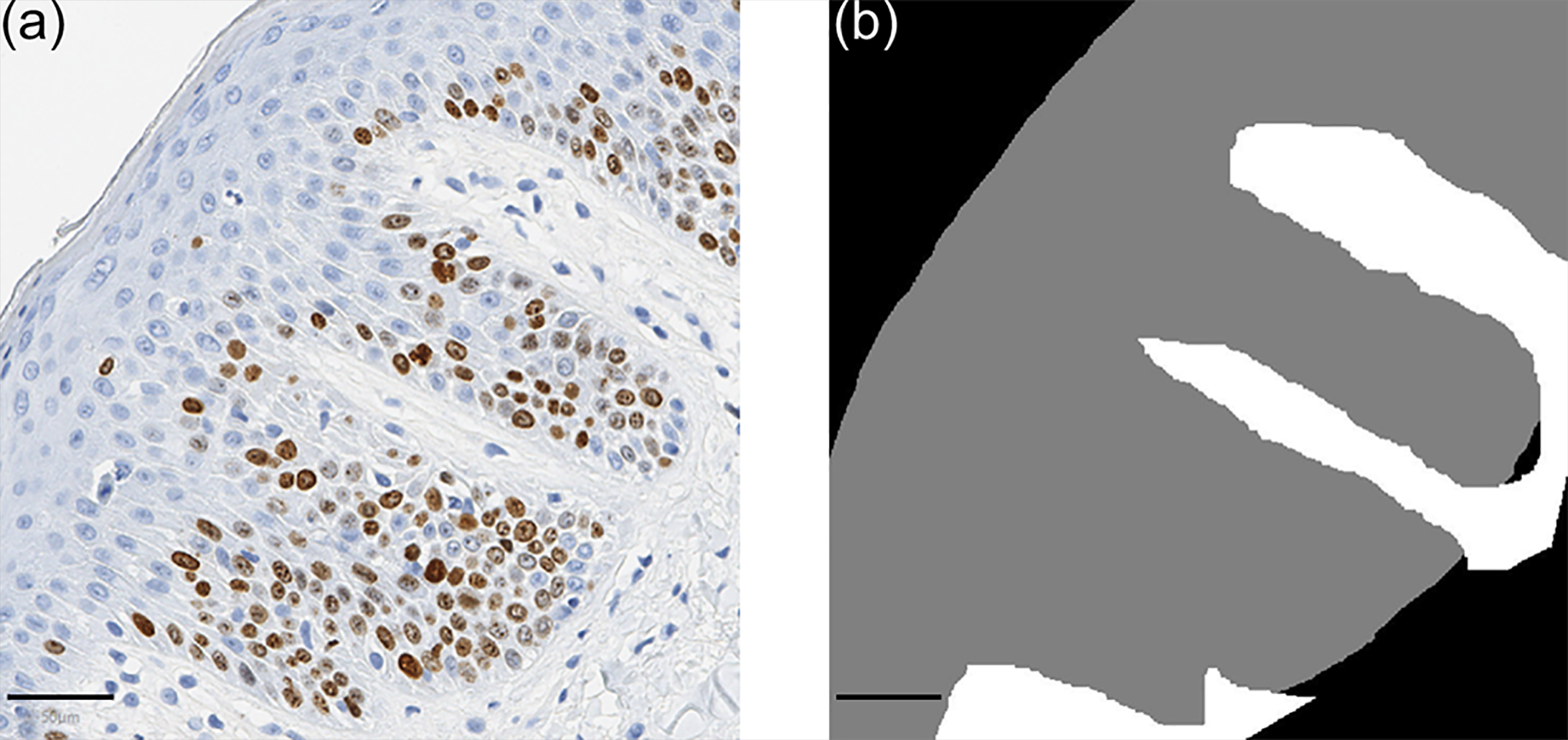

Supplement: Supplementary file 2 — Figure S2 [file JDV-37-605-s005.tiff]

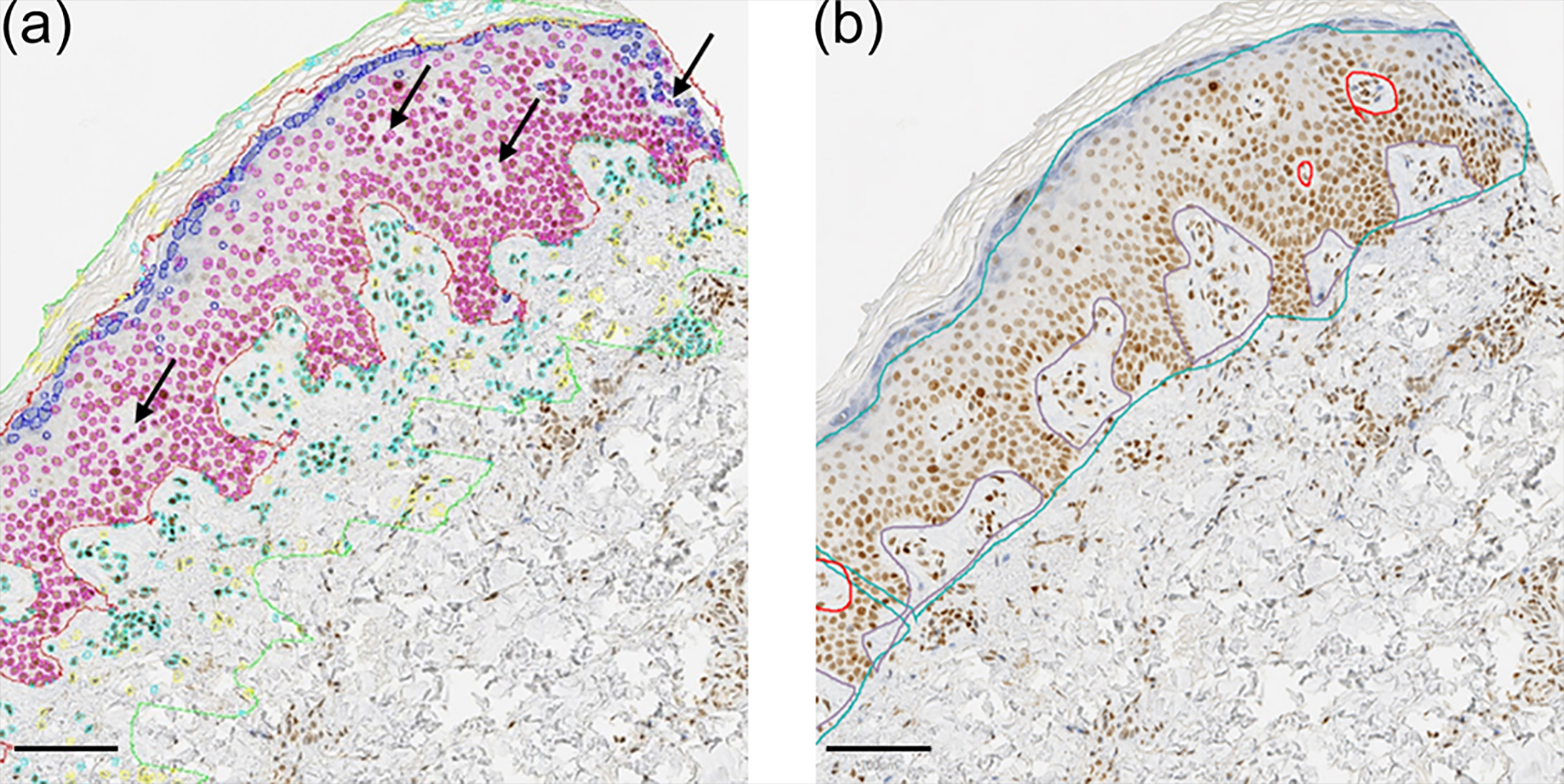

Supplement: Supplementary file 4 — Figure S4 [file JDV-37-605-s004.tiff]

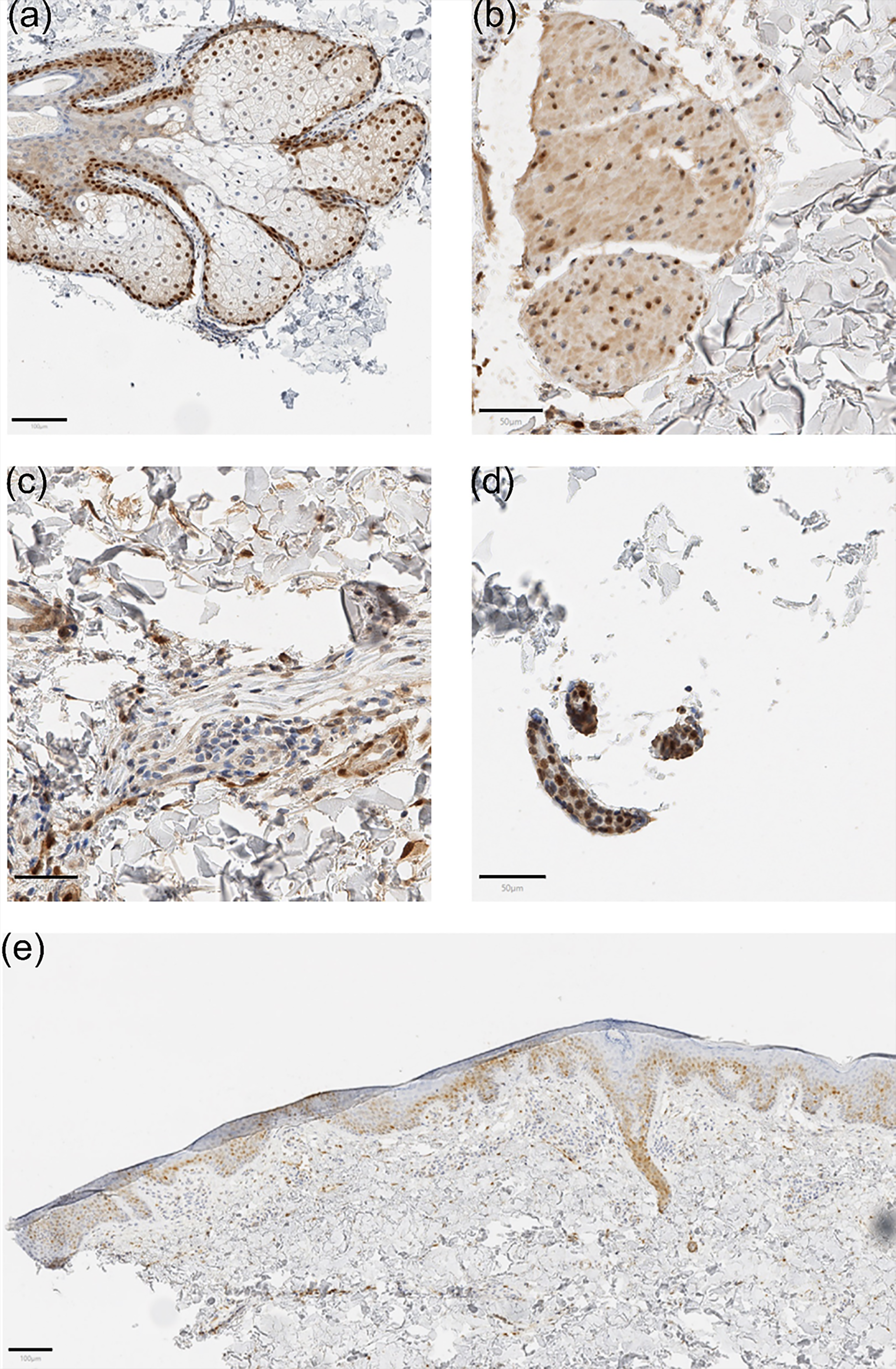

Supplement: Supplementary file 5 — Figure S5 [file JDV-37-605-s001.tiff]

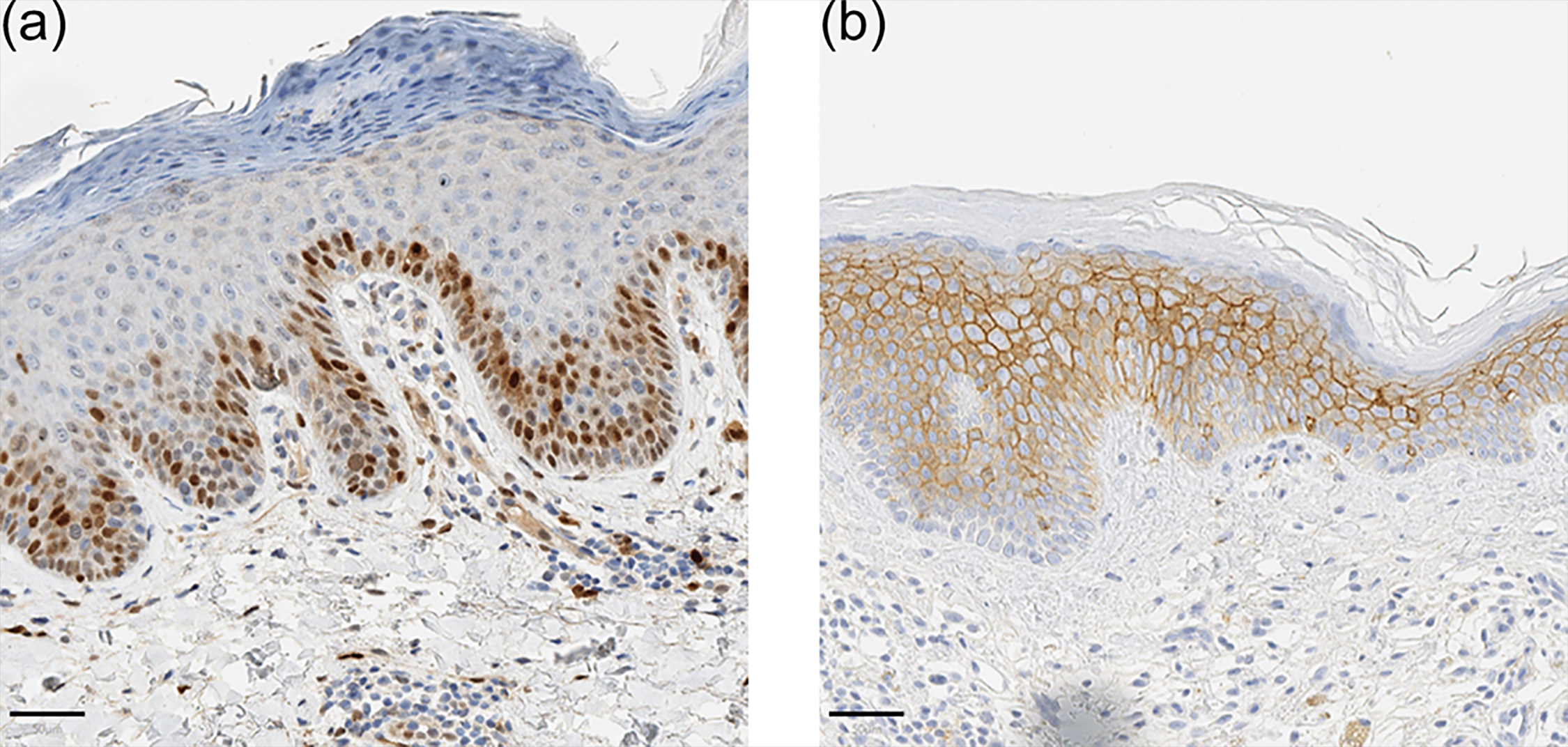

Supplement: Supplementary file 6 — Figure S6 [file JDV-37-605-s006.tiff]
