# Supplementary material for: An open source pipeline for quantitative immunohistochemistry image analysis of inflammatory skin disease using artificial intelligence
Source: J Eur Acad Dermatol Venereol. 2022 Dec 3;37(3):605–14. doi: 10.1111/jdv.18726 (PMC10947200; doi:10.1111/jdv.18726)

Supplementary Figure 3

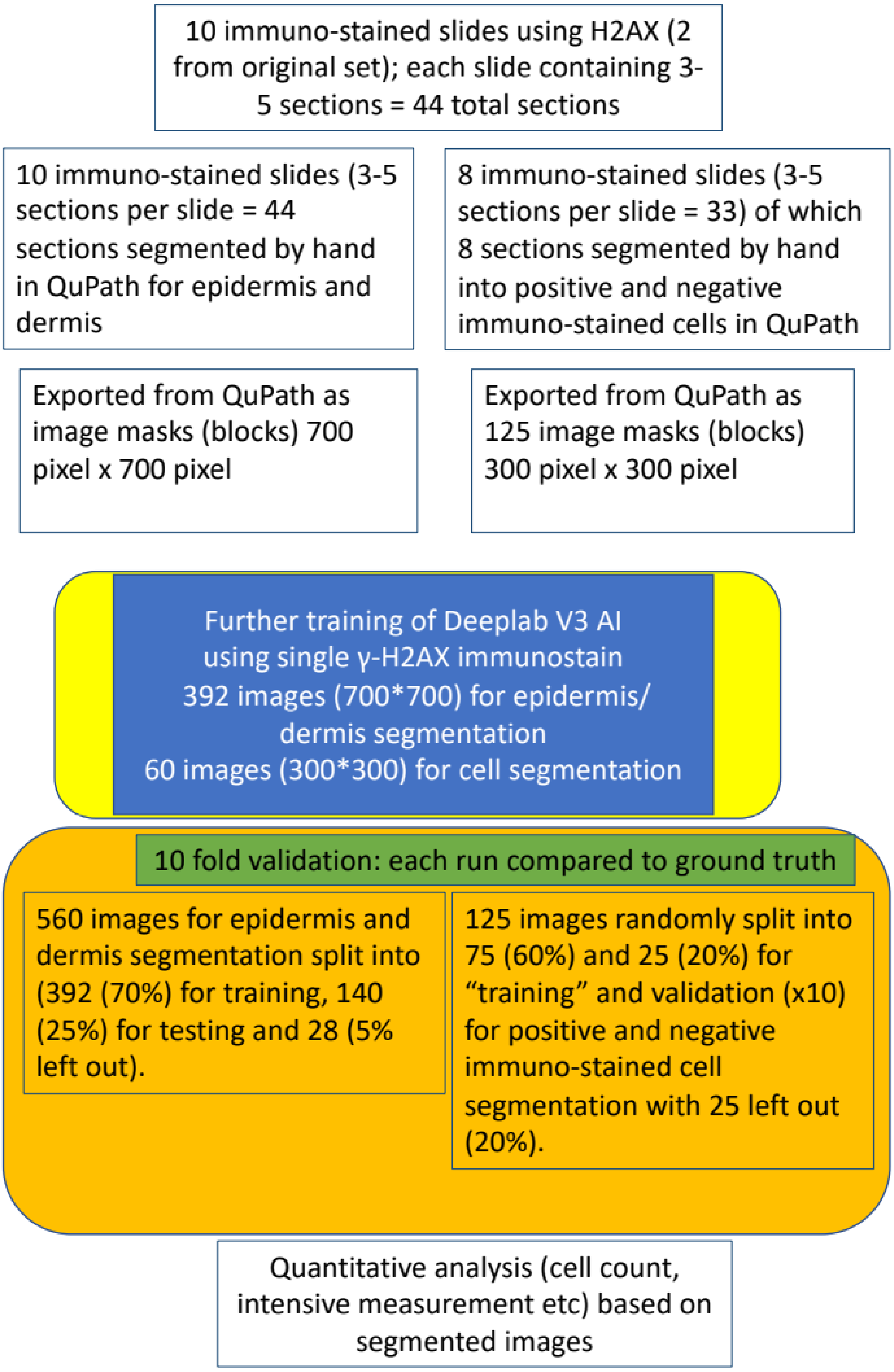

Supplement: Supplementary file 2 [file JDV-37-605-s003.docx]
